# Dietary patterns and risk of mild cognitive impairment among Chinese elderly: A cross-sectional study

**DOI:** 10.1371/journal.pone.0235974

**Published:** 2020-07-13

**Authors:** Xiangni Su, Jieqiong Zhang, Wenchen Wang, Chunping Ni, Shanbo Hu, Pei Shao, Cuicui Li, Yan Hua, Hongjuan Lang, Yi Wan

**Affiliations:** 1 Department of Nursing, Fourth Military Medical University, Xi’an, Shaanxi, China; 2 Department of Health Services, Fourth Military Medical University, Xi’an, Shaanxi, China; 3 Department of Neurology, Air Force General Hospital, Beijing, China; 4 Department of Sports Medicine, Honghui Hospital, Xi'an Jiaotong University, Xi'an, Shaanxi, China; University of Cádiz, SPAIN

## Abstract

**Objective:**

To investigate the relationship between food patterns and mild cognitive impairment (MCI) among Chinese elderly to provide evidence for risk prevention and control of MCI among elderly population.

**Methods:**

Between February 2017 to October 2018, a stratified multistage cluster sampling method was used to select participants from 760 communities of six districts in Xi’an, China, for 49-item food frequency questionnaire survey. A total of 2311 participants aged 60 to 88 years were included in the study with 444 (19.2%) participants of MCI among Chinese community-dwelling elderly adults. Food patterns associated with risk of dementia were assessed by using a reduced rank regression (RRR) analysis, and the multivariate linear regression was used to test trends of risk factors across scores for the food pattern.

**Results:**

Four dietary patterns were extracted which explained 88.65% of the total variation in food intakes. Furthermore, the food pattern 1 (FP1) accounted for 60.25% of the total variation of all responsible variables and as a target dietary pattern in the study, which was related with high intake of legumes, vegetables, fruits, milk and dairy products, nuts and a low intake of noodles and cereals (*p*<0.05). Multivariate linear regression analysis showed that participants with a high score for FP1 had higher direction, memory and language function and FP1 can improve the ability of cognitive function (*p*<0.05).

**Conclusion:**

The FP1 of Chinese dietary patterns was significantly correlated with higher cognitive function which can reduce the risk of MCI among Chinese elderly.

## Introduction

Cognitive impairment is common among older population worldwide which become an increasingly important public health issue [1]. There are nine million Chinese people suffer from dementia, with an incidence of 8–10% among people over 65 years of age [2]. In China, the total cost of dementia for 2010 and 2030 represent about 7.8% of estimated US $604 billion global cost in 2010 and 10% of forecasted US $1110 billion global cost in 2030 [2]. Mild cognitive impairment (MCI) is an intermediate stage between normal cognitive function and dementia among the elderly [3]. Numerous studies indicated that MCI can be regarded as a significantly high risk of dementia, and some even shown that 60–100% of MCI patients will progress to dementia over 5–10 years [4, 5]. Currently, there are no effective pharmacological treatments for these devastating and disabling conditions, which emphasize the key role of preventive strategies. Hence, it is critical to identify modifiable risk factors to retard the progression of MCI with aging, and thus prevent or delay the occurrence of dementia.

There is compelling evidence of the role of diet on cognitive function. Poor nutritional status or dietary pattern is associated with cognitive and functional decline in non-MCI and also predicts is a faster rate of cognitive impairment in those with MCI individuals [6]. An increasing body of epidemiological evidence suggests that elevated saturated fatty acids (SFA) could have negative effects on age-related cognitive decline (ARCD). However, high fish consumption, and high intake of monounsaturated fatty acids (MUFA) and polyunsaturated fatty acids (PUFA), particularly n-3 PUFA reduces the risk of MCI [7].

Various studies revealed that adherence to the Mediterranean diet (MeDi), characterized by high intake of whole grains, legumes, vegetables and fruits with moderate consumption of alcohol and a high monounsaturated-to-saturated fat ratio, has been related to reduce MCI and dementia [8]. Some studies showed that specific dietary pattern interventions such as dietary approach to systolic hypertension (DASH) and Mediterranean-DASH diet intervention for dementia delay may also produce effective preventive approaches [9]. However, most of these findings have been investigated in Western individuals, and evidence from Asian individuals is limited. Otherwise, because of dietary available and cultural norms, there can be significant difference in the foods typically consumed by different individuals. Therefore, these patterns are not suitable for Chinese dietary habits especially in elderly. In China, elderly traditional dietary patterns were characterized by high proportion of rice or noodle-based food and animal-based food, lack of green vegetables and fresh fruits [10]. These traditional Chinese dietary patterns were positive associated with cognitive decline, hypertension, and overweight [11, 12].

Furthermore, we hypothesized that there is another food pattern like Mediterranean dietary pattern for westerner that would be equally or more effective for Chinese elderly individuals. Nevertheless, potential relationships between the Chinese’ elderly diet patterns and MCI have not been reported. Therefore, the study aimed to estimate dietary factors associated with the development of MCI in community-dwelling Chinese elderly individuals, which will be of great significance to guide people to prevention and control of dementia.

## Methods

### Study participants

A stratified multistage cluster sampling method was used to include participants between February 2017 to November 2018 in Xi’an, China, according to the sequence of districts, streets and communities. Finally, a total of 16 communities were selected in the study. All the elderly participants aged 60 years and older who were living in the selected communities were included for the questionnaire survey. Participants were eligible for the study if they were 60 years of age or over; who were living the selected communities; who were able to communicate with investigators normally. Participants were excluded if they had a known diagnosis of dementia; who had history of schizophrenia; who had lived in the selected community for less than five years or were not permanent residents.

The study was approved by the Ethics Committee of the Fourth Military Medical University, and all participants provided informed consent prior to participation.

### Mild cognitive impairment

Two nurses and neuropsychology professors assessed all participants at their communities for cognitive impairment. The study adopted the MCI diagnostic criteria from Petersen definitions with two modifications [13]: (Ⅰ) A combination of activities of daily living (ADL) and instrumental activities of daily living (IADL) was used to define intact ADL; (Ⅱ) ➀ memory complaints (either self-reported or reported by family members or caregivers); ➁ Continued normal cognitive function, as assessed through the mini-mental state examination (MMSE); ➂ essentially intact ADL and IADL; no dementia (≤ 1.5 S.D. form norm), as evaluated by the Chinese version of Dementia Rating Scale (CDRS); ➃ no abnormal memory impairment for age.

A brief neuropsychological battery was used to assess cognitive function in the participants with MCI. In the study, cognitive function was assessed by the Chinese version of the mini-mental state examination (MMSE) [14]. It has been reported that when education is considered, the Chinese version of the MMSE indicates MCI if the score is ≤17 for illiterates, ≤20 for primary school graduates (≥6 years of education), and ≤24 for junior school graduates or above (≥9 years of education) [14,15]. The Chinese version of the MMSE has high sensitivity (90.8%) and specificity (93%) in the identification of dementia among the Chinese population [16].

The Chinese version of the Mattis Dementia Rating Scale (CDRS) was used to assess the general cognitive status, which included measurements of attention, initiation and perseveration, construction, conceptualization and memory [17]. The CDRS cut-off scores for dementia in the Chinese population according to education level, illiteracy, primary school graduates (≥6 years of education), junior school graduates (≥9 years of education) revealed a sensitivity of 85%, 94%, 94%, respectively, and a specificity of 90%, 94%, 92%, respectively [18].

### Functional status

An individual’s functional status was assessed using six items on the activities of daily living (ADL) scale and eight items instrumental activities of daily living (IADL) [19]. Both scales had been widely used and were subsequently adapted for use in Chinese participants [20]. The Chinese version of the ADL scale includes 20 items, and its reliability and validity had been verified among older Chinese participants [21], which had been reported previously.

### Questionnaire survey and measurement indicators

A face-to-face interview using a semi-quantitative food frequency questionnaire (FFQ), was conducted at homes of the participants, and anthropometric measurements were taken using World Health Organization (WHO) standard protocol [22] at community health service centers, by trained staff for both cases.

The FFQ was based on the Chinese Food Guide which is extensively used for dietary guidelines for Chinese residents [23]. FFQ has been validated to be a reasonable tool to assess relationships between dietary habits and MCI [24]. All participants filled out the FFQ about the frequencies of each food intake. The average food intake per day was calculated from the weekly frequency of intake of various foods and the amount (quantity) of each food portion. All completed questionnaires were checked by three research nurses and missing or unclear responses were clarified when the participants attended their physical assessment. Each nutritional element was adjusted for energy intake by using the nutrient density method [25].

According to the WHO recommendation [22], weight was recorded to the nearest 0.1 kg without shoes and in light clothing on a calibrated beam scale. Height was determined to the nearest 0.1 cm without shoes using the SECA stadiometer. Body mass index (BMI) was calculated as weight (kg) divided by height (m) squared [12]. Systolic and diastolic blood pressure (SBP and DBP) were recorded as means of three reads after 10 min of rest using mercury sphygmomanometers with appropriated cuff sizes. Participants with hypertension or diabetes were diagnosed by hospital or current use of antihypertensive or treatment diabetes drugs. A history of stroke was defined as a pre-existing sudden onset of nonconvulsive and focal neurologic deficit that persisted >24 h on the basis of all available clinical data.

### Statistical analyses

All completed questionnaires were anonymized with identity number. Double data entry was conducted by using the Epidata Version 3.1. (EpiData Association, Odense, Denmark). Computer and manual checks ensured accurate data coding. Data were analyzed using the SPSS 17.0 for Windows (SPSS Inc., Chicago, IL, USA) and the SAS software package (version 9.2; SAS Institute, Cary, NC, USA).

The association between the 49-food groups and the four dietary patterns was analyzed by factor loadings, which denote the relationship between the foods and the patterns. Food groups with factor loadings ≥0.2 were considered positive contributors to the patterns, and foods with factor loadings up to -0.2 were negative contributors to the patterns.

Food patterns associated with risk of MCI were assessed by using a reduced rank regression (RRR) analysis [26], which is a statistical approach that extracts successive linear combinations of food groups (predictor variables) that explain as much as possible the variation in the response variables that are hypothesized to be associated with the outcome [27]. The number of patterns generated will be the same as the number of predictor variables considered in the model. In our study, 49 food groups (g/d) were considered as the predictor variables. The PROC PLS procedure with the RRR method option was adopted to derive the food patterns, using the SAS Statistical Software Version 9.2. The residuals of the models were normally distributed.

A total of four nutrients were selected as risk or preventive factors for MCI: saturated fatty acids (SFA), monounsaturated fatty acid (MUFA) polyunsaturated fatty acid (PUFA), and Vitamin C [28–30]. Four dietary patterns were extracted by using the RRR. These nutrients were known or suspected to confer risk of or protection against cognitive impairment and were variables with *P* <0.2 in the univariate analyses regarding their intakes and risk of the development for MCI. Pearson’s correlation coefficients were calculated in food groups, nutrients and scores for the extracted food pattern. The scores of the food pattern were categorized in quartiles. The multivariate linear regression analysis was used to test trends in mean values or frequencies of risk factors across scores for the food pattern. Two-sided *P*<0.05 was considered statistically significant for all analyses.

## Results

**Fig 1:**
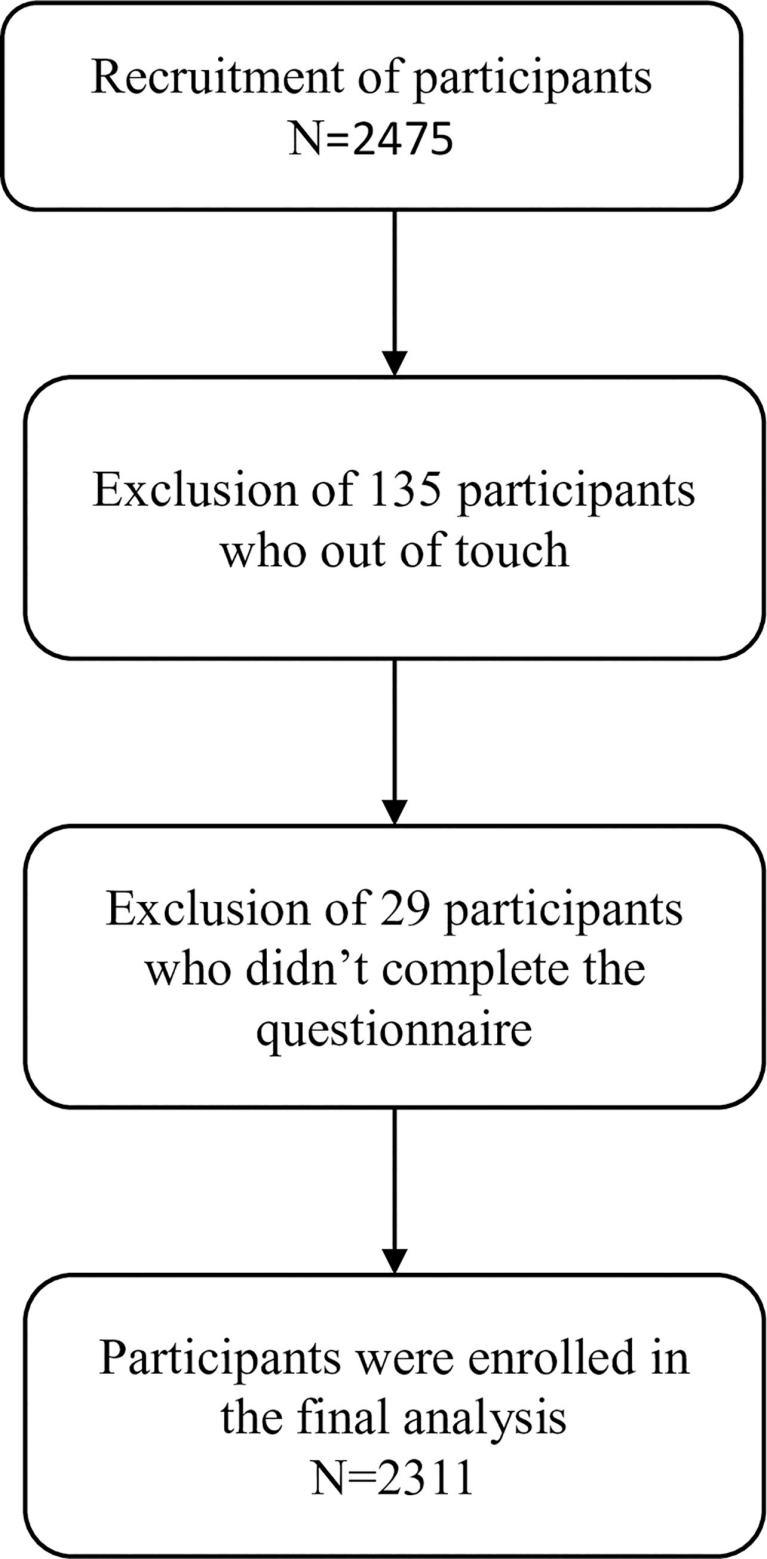
Flow chart of the study participants.

After the exclusion of 135 participants who out of touch, and 29 participants who didn’t complete the questionnaire, 2311 participants were enrolled in the study with response rate of 93.4%, of which 444 (19.2%) participants were diagnosed with MCI (Fig 1). Among the included participants, 62.7% were women and 37.3% were men. The mean age of the individuals was 73.3±7.9 years. The prevalence of diabetes and hypertension were 12.3% and 43.4%, respectively. In total subjects, 13.9% and 8.2% of subjects had smoking habits and alcoholic drinks. Intake of total energy, protein, carbohydrates were 9464 ± 2737 kj/d (which was 2261 ± 654 Kcal/d), 72.6 ± 27.3 g/day, 304.5 ± 121.2 g/day, respectively (Table 1).

**Table 1:** Demographic and daily dietary intake of selected food groups among elderly (*n* = 2311).

Variables	Values^a^
Age (years)	73.3±7.9
Gender	
Males	862 (37.3)
Females	1449 (62.7)
Smoking	321 (13.9)
Drinking	190 (8.2)
Diabetes	284 (12.3)
Hypertension	1003 (43.4)
MMSE	24.32 ± 5.13
BMI	28.31±1.63
Energy (KJ/day)	9464 ± 2737
Proteins (g/day)	72.6 ± 27.3
Carbohydrates (g/day)	304.5 ± 121.2
MUFA	45.2 ± 10.6
PUFA	7.6 ± 2.7
SFA	21.6 ±6.9
Total fibers (g/day)	0.5±12.5
Insoluble fibers (g/day)	22.9 ± 6.9
Soluble fibers (g/day)	9.0 ± 4.1

^*a*^. Values were presented as mean ±S.D. or frequency (percentage) as suitable. MMSE: Mini-Mental State Examination (MMSE); BMI: Body Mass Index; MUFA: Monounsaturated Fatty Acid; PUFA: Polyunsaturated Fatty Acid; SFA: Saturated Fatty Acid

In the study, RRR was used to extract four different dietary patterns to identify the main food pattern: SFA, MUFA, PUFA, and vitamin C, which explained 80.71% of variation of all nutrients. The proportion of scores for food pattern 1 (FP1) was 70.60% (56.98/80.71) which was the highest among the four food patterns (the scores of FP2, FP3, FP4 were 22.62%, 0.46%, 0.56%, respectively). Pearson’s correlation coefficients of food patterns greater than 0.50 were defined as a target food pattern, with only FP1 being selected in our study (Table 2).

**Table 2:** Pearson’s correlation coefficients and explained percentage between nutrients (dependent variables) and extracted food patterns (independent variables) (*n* = 2311).

Nutrients	Food patterns				Total
	1	2	3	4	
SAF	0.52 (26.02) *	0.43 (20.16) *	0.06 (0.35)	0.18 (1.23) *	47.76
MUFA	0.82 (67.54) *	0.52 (29.04) *	-0.64 (0.39)	-0.04 (0.17)	97.50
PUFA	0.76 (65.29) *	0.44 (23.74) *	0.12 (0.45) *	0.05 (0.28)	89.76
Vitamin C	0.83 (69.08) *	0.32 (17.52) *	0.08 (0.64)	0.10 (0.57)	87.81
Explained proportion of variation of all nutrients	56.98	22.62	0.46	0.56	80.71

Food patterns were derived by RRR analysis.

* *P*< 0.001. SFA: Saturated Fatty Acid; MUFA: Monounsaturated Fatty Acid; PUFA: Polyunsaturated Fatty Acid.

Factor analysis was used to identify the correlations between FP1 and food group and four independent variables (Table 3), which also represented the magnitude and direction of each food group’s contribution to scores for FP1. Food groups with positive value of factor loading more than 0.2 were legumes, green and other vegetables, fruits and fruit juices, milk and dairy products and nuts; however, the food with a factor loading less than -0.2 was noodles and cereals.

**Table 3:** Factor loadings of food groups associated with food pattern 1 and correlation coefficients between food groups and four nutrients (*n* = 2311).

		Correlations between food groups and 4 response variables			
Food group	Factor loading (FP1)	SAF	MUFA	PUFA	Vitamin C
Noodles and cereals	-0.42	-0.47	-0.48	-0.52	-0.03
Steamed buns	0.12	0.30	0.13	0.13	-0.02
Legumes	0.43	0.06	0.30*	0.73*	0.02
Pickles	0.04	-0.07	-0.09	-0.12	0.33*
Green vegetables	0.43	-0.01	0.07	0.14	0.75*
Other vegetables	0.37	-0.01	0.04	0.06	0.76*
Fruits and fruit juices	0.21	-0.01	-0.02	-0.03	0.57*
Fish and shrimp	0.19	-0.06	0.18*	0.23	-0.07
Meat	0.01	0.37*	0.12	-0.16	0.02
Egg	0.15	0.32*	0.37*	0.06	0.04
Milk and dairy products	0.39	0.64*	0.29*	0.06	-0.03
Fats and animal oils	0.12	0.43*	0.62*	0.24*	0.08
Plant oils	0.10	0.41*	0.57*	0.30*	0.10
Sugar and desserts	-0.12	0.01	-0.05	-0.13*	-0.01
Alcoholic drinks	-0.14	-0.15	-0.24*	-0.18*	-0.22*
Nuts	0.36	0.07	0.37*	0.81*	0.07
Salt	-0.01	-0.06	-0.01	-0.16*	0.25

Pearson’s correlation coefficients were used between FP1 and food group and nutrients

*P<0.001.

Logistic regression analysis showed that participants with a high score for FP1 were likely to be women and more likely to have hypertension, smoking habits, BMI and MCI (Table 4).

**Table 4:** Demographic characteristics of the study individual by quartiles of score for FP1 (*n* = 2311).

Variables	Scores for FP1				
	Q1 (n = 578)	Q2 (n = 578)	Q3 (n = 578)	Q4 (n = 578)	*P*
	< -0.81	-0.81 to -0.05	-0.07 to 0.84	≥0.84	
Scores for FP1 ^a^	-1.7	-0.3	0.4	1.6	<0.001
Age (year) ^b^	70±6.9	71±5.4	71±5.5	70±6.8	0.71
Women (%)	44.3	56.9	60.2	77.3	<0.001
Education (%)					
≤6 years	9.8	11.1	12.3	7.6	0.13
7~12 years	87.9	80.3	78.6	81.2	0.46
≥13 years	2.3	5.6	9.1	11.2	0.01
SBP (mmHg) ^b^	140±24	137±24	139±23	138±22	0.56
DBP (mmHg) ^b^	78±12	76±10	77±12	78±11	0.43
Hypertension (%)	36.0	37.7	41.2	45.9	<0.001
Diabetes (%)	6.7	5.8	6.6	6.3	0.07
BMI (kg/m^2^)	22.1±4.1	22.5±4.2	23.3±4.0	24.7±3.8	<0.001
History of stroke (%)	4.5	4.7	4.1	4.4	0.82
Smoking (%)	4.3	4.9	5.8	7.0	<0.001
MMSE	26.2±3.1	23.2±3.7	22.2±3.0	21.2±3.6	<0.001

General linear model and Logistic regression analysis were used to test in mean values of risk factors across scores for the dietary pattern and in frequencies of risk factors across scores for the food pattern, individually.

^a^ All values are medians.

^b^ Mean ± SD (all such values). MMSE: Mini-Mental State Examination (MMSE); SBP: systolic blood pressure; DBP: diastolic blood pressure; BMI: body mass index.

Multivariate linear regression analysis showed that participants with a high score for FP1 were likely to have higher direction, memory and language function and FP1 can improve the ability of cognitive function (Table 5).

**Table 5:** The relationship between domains of MMSE and the quartiles of score for FP1.

Domains of MMSE	Score for food pattern 1				
	Q1	Q2	Q3	Q4	*P*
Direction	1	1.43	1.81	2.60	<0.001
Memory	1	1.21	1.42	2.97	<0.001
Concentration and attention	1	1.29	0.83	0.71	0.209
Immediate and delayed memory	1	1.05	1.18	1.34	0.092
Language	1	1.17	1.28	1.59	0.016

The data in the table represent standardized partial regression coefficients by multivariate linear regression analysis.

## Discussion

The study showed that the FP1 can reduce and delay the prevalence of MCI, especially in direction, memory and language function of cognitive. The FP1 was characterized by high intake of legumes, green and other vegetables, fruits and fruit juices, milk and dairy products and nuts and lower level of noodles and cereals. However, the food pattern of community-dwelling Chinese nonagenarians and centenarians includes low intake of fresh fruits, vegetable and milk and dairy products as well as high intake of noodles and cereals, which may contribute to cognitive decline [31]. The results of our study will provide valuable evidence for the establishment of preventive strategies against MCI through lifestyle modification among the elderly Chinese population.

Mio et al [28] used seven nutrients as response variables: SFA, MUFA, PUFA, vitamin C, potassium, calcium, and magnesium. It was found that the extracted dietary pattern was positively associated with high intake of soybeans and soybean products, green vegetables, fruit, milk and dairy products, nuts and fish, and negatively correlated with fats and oil, sugar and confectioneries, salt and drinks, and individuals with great adherence to this food pattern had lower risk of MCI. Study had found the beneficial effect of the MeDi pattern on decreasing the risk of MCI and revealed that higher intakes of green vegetables, legumes, fruit, milk and dairy products, nuts and fish were linked to lower risk of MCI [32]. Because Japanese diet is similar to Chinese, similar findings were also observed in our study in spite of significant difference of dietary custom among study participants.

In the study, fruits and fruit juices was positively related with higher scores for FP1, which related to lower risk of MCI, so intake of vitamin C was included in the RRR analysis as protection factor for cognitive function. Similar result was also reported in several Asian cohorts, consumption of diets featuring of vitamin C is associated with reduced risk of MCI [33]. The relationship between fruits and vegetables and cognitive function may be attribute to beneficial effect of antioxidants and vitamins C, D, E rich in these foods. Greater antioxidant intake could prevent age-related cognitive dysfunction because brain tissue contains low levels of endogenous antioxidants, which is particularly vulnerable to free-radical damage [34]. Furthermore, a diet rich in fruits and vegetables contains a high fiber consumption that positively influences gut microbiota, which might also be associated with better cognitive health and needs to be further studied [35].

Milk and other dairy products may contribute to the prevention of physical and cognitive impairment [36]. Our study found that milk and dairy products intake was positively correlated with higher scores for FP1, which had beneficial effect on cognitive function. However, in older Japanese individuals a significant inverse relation between dairy intake and the development of AD was found [36]. A longitudinal study suggested no significant association between consumption of dairy products and MCI in French women [37]. Petruski-Ivleva et al showed that a milk intake >1 glass/d at midlife associated with a greater rate of cognitive decline over a 20-y period. However, none of these studies had evaluated the effects of vitamin D. Full-fat dairy products contain vitamin D, which relates to neuroprotective, antioxidant, and anti-inflammatory effects [38].

The study revealed that PUFA improved specific cognitive domains and cognitive-related outcomes in MCI, mild-to-moderate dementia [39], and AD. Our study also found that high MUFA and PUFA were significantly associated with a higher cognitive function. As it well-know, DHA is a key nutritional n-3 PUFA and needs to be supplied by the human diet. DHA is found in significant amounts in the retinal and neuronal cell membranes due to its high fluidity [40]. MUFA and PUFA played an important role in ensuring a healthy ageing, by thwarting macular degeneration, Alzheimer’s disease, and other brain disorders at the same time as enhancing memory and strengthening neuroprotection in general [41]. Gu et al found that n-6 and n-3 PUFA could decrease the incidence of cognitive impairment through influence on neuronal membrane integrity [42]. Animal studies also found that greater intake PUFA may protect cognitive function by decreasing the effect of amyloid deposition within the brain [43].

Furthermore, our study showed a negative correlation between noodles and cereals and FP1. Noodles always play a great part of the Chinese daily diet, especially in northwest of China. This relationship may attribute to an imbalance in food intake rather than any harmful effects of noodles and cereals itself. In the present investigation, a number of elderly individuals consumed a high intake of noodles and cereals, which may lead to lower intake of green vegetable or foods favorable for the prevention of cognitive function.

The study also had some limitations. Firstly, the cross-sectional design of the study cannot provide evidence of any causal relationships between food patterns and MCI. It must also consider the possibility of reverse causality, such that cognitive decline may have already been presented and this may have resulted in a diminished ability to consume healthy foods. Secondly, the intake of dietary nutrients information was derived from the FFQ, a semi-quantitative food-frequency questionnaire, which can’t be fully valid for ranking subjects according to food and nutrient intake as in the present study. Thirdly, study participants were primarily of northwest China elderly and any generalizability to other areas should be performed with caution. However, the study was specifically designed to investigate risk factors for MCI. The population-based design reduced selection bias and the comprehensive evaluation of participants for MCI by two independent evaluators increased the internal validity of the findings.

Above all, the study indicated a dietary pattern which could improve cognitive impairment among elderly in northwest of China, and individuals with this food pattern had negative relationship with risk of cognitive impairment. Further study is suggested to focus on identifying strategies to help individual maintaining balanced diet, which could reduce the risk for the development of dementia.

## Supporting information

S1 Data (SAV)
